# Analysis of a new strain of *Euphorbia mosaic virus *with distinct replication specificity unveils a lineage of begomoviruses with short Rep sequences in the DNA-B intergenic region

**DOI:** 10.1186/1743-422X-7-275

**Published:** 2010-10-19

**Authors:** Josefat Gregorio-Jorge, Artemiza Bernal-Alcocer, Bernardo Bañuelos-Hernández, Ángel G Alpuche-Solís, Cecilia Hernández-Zepeda, Oscar Moreno-Valenzuela, Gustavo Frías-Treviño, Gerardo R Argüello-Astorga

**Affiliations:** 1 Instituto Potosino de Investigación Científica y Tecnológica, A.C., Camino a la Presa San José, 78216 San Luís Potosí, SLP, México; 2 Universidad Autónoma Agraria Antonio Narro. Departamento de Parasitología Agrícola. Bellavista, C.P. 25315, Saltillo, Coahuila, Mexico; 3 3Centro de Investigación Científica de Yucatán, A.C., Mérida, Yucatán, México

## Abstract

**Background:**

*Euphorbia mosaic virus *(EuMV) is a member of the SLCV clade, a lineage of New World begomoviruses that display distinctive features in their replication-associated protein (Rep) and virion-strand replication origin. The first entirely characterized EuMV isolate is native from Yucatan Peninsula, Mexico; subsequently, EuMV was detected in weeds and pepper plants from another region of Mexico, and partial DNA-A sequences revealed significant differences in their putative replication specificity determinants with respect to EuMV-YP. This study was aimed to investigate the replication compatibility between two EuMV isolates from the same country.

**Results:**

A new isolate of EuMV was obtained from pepper plants collected at Jalisco, Mexico. Full-length clones of both genomic components of EuMV-Jal were biolistically inoculated into plants of three different species, which developed symptoms indistinguishable from those induced by EuMV-YP. Pseudorecombination experiments with EuMV-Jal and EuMV-YP genomic components demonstrated that these viruses do not form infectious reassortants in *Nicotiana benthamiana*, presumably because of Rep-iteron incompatibility. Sequence analysis of the EuMV-Jal DNA-B intergenic region (IR) led to the unexpected discovery of a 35-nt-long sequence that is identical to a segment of the *rep *gene in the cognate viral DNA-A. Similar short *rep *sequences ranging from 35- to 51-nt in length were identified in all EuMV isolates and in three distinct viruses from South America related to EuMV. These short *rep *sequences in the DNA-B IR are positioned downstream to a ~160-nt non-coding domain highly similar to the CP promoter of begomoviruses belonging to the SLCV clade.

**Conclusions:**

EuMV strains are not compatible in replication, indicating that this begomovirus species probably is not a replicating lineage in nature. The genomic analysis of EuMV-Jal led to the discovery of a subgroup of SLCV clade viruses that contain in the non-coding region of their DNA-B component, short *rep *gene sequences located downstream to a *CP*-promoter-like domain. This assemblage of DNA-A-related sequences within the DNA-B IR is reminiscent of polyomavirus microRNAs and could be involved in the posttranscriptional regulation of the cognate viral *rep *gene, an intriguing possibility that should be experimentally explored

## Background

The members of the family *Geminiviridae*, one of the two largest natural groups of plant viruses, are characterized by a circular, single-stranded DNA (ssDNA) genome encapsidated within virions whose morphology is unique in the known virosphere, consisting of two joined, incomplete T = 1 icosahedra [1,2]. Geminiviruses are classified into four genera, based on their genome organization, plant host range, and insect vector. Members of the most diversified genus, *Begomovirus*, are transmitted by the whitefly *Bemisia tabaci *(Hemiptera; Aleyrodidae), infect a wide range of dicotyledonous plant species, and have either monopartite or bipartite genomes [3]. In recent decades, these viruses have emerged as major threats to food and fiber crop production throughout the world, apparently as a result of a great increase in vector population densities, expansion of crop monocultures, transport of plant materials between geographically distant regions, and introduction of foreigner whitefly biotypes [4,5].

Approximately 200 species of begomoviruses are currently known, grouped into two major lineages based on their genomic sequences: the Old World (OW; Europe, Africa, the Indian subcontinent, Asia, and Australasia) and the New World (NW; the Americas) begomoviruses [6,7]. The OW begomoviruses have either monopartite or bipartite genomes, while all NW begomoviruses (for simplicity, NW-Beg) have two genomic components, known as DNA-A and DNA-B. The DNA-A component of NW-Beg has one open reading frame in the virion sense (*AV1 *or *cp *gene) encoding the coat protein, and four overlapped ORFs in the complementary sense (*AC1 *or *rep *gene, *AC2 *or *trap *gene, *AC3 *or *ren *gene, and *AC4*) that encode proteins involved in DNA replication, regulation of viral gene expression and suppression of host-defense responses [1,8]. The DNA-B component contains only two ORFs, one in the virion sense (*BV1 *or *nsp *gene) and other in the complementary sense (*BC1 *or *mp *gene), encoding proteins involved in intra- and intercellular movement of the virus [9,10]. The two genomic components are very different in overall nucleotide sequence, with the exception of a ~180-nt segment of the intergenic region (IR) displaying high sequence identity, termed the "common region" (CR). This region includes several repeated sequences (5 to 8-nt in length) called "iterons", which are closely associated to a ~30-nt conserved element that has the potential to form a hairpin structure that harbors in its apex the invariant nonanucleotide 5'-TAATATTAC- 3' [1]. Both the iterons and the conserved nonanucleotide in the hairpin element are functional targets for Rep, the virus-encoded protein that initiates the DNA replication by a rolling-circle (RCR) mechanism. Rep recognizes and binds specifically to the iterons and subsequently introduces a nick into the invariant nonanucleotide to initiate the RCR process [11,12].

The NW-Beg have radiated to a great extent since its arrival to the American continent, and several secondary lineages or "clades" have been identified in phylogenetic studies [6,13,14]. The most atypical of the NW-Beg clades is the one named after the *Squash leaf curl virus *(SLCV) that encompasses more than 15 viral species distributed from Southern EUA to Brazil [7,13]. Members of the SLCV clade are differentiated from other NW-Beg by two main features: 1) the number and arrangement of the iterons in their replication origin, that are distinctive, and 2) the N-terminal domain (i.e., residues 1 to 150) of their Rep proteins display low aa sequence identity (< 50%) with proteins encoded by typical NW-Beg, lacking several amino acid motifs which are conserved in both NW- and OW- begomovirus Rep proteins [[15-17]; unpublished data].

Among the earliest recorded members of the SLCV-clade is *Euphorbia mosaic virus *(EuMV), which was associated with symptomatic *Euphorbia heterophylla *plants throughout the Caribbean basin and the tropical Americas since the 1970's [18,19]. However, its molecular characterization was not carried out until 2007, when the complete genome sequence of EuMV-YP, the isolate associated with the former plant host in the Yucatan Peninsula of Mexico, was reported [20]. Complete DNA-A sequences from two additional EuMV isolates were available at GenBank at that time, one from Puerto Rico (EuMV-PR) and the isolate whose complete sequence is now reported here, from Jalisco, Mexico (EuMV-Jal). According to their full-length DNA-A sequence identity, the EuMV isolates were classified into two different strains, simply termed "A" and "B". The first strain was represented by EuMV-YP and EuMV-PR, while EuMV-Jal was the only member of the "B-strain" [7]. However, the recently described EuMV-JM, from Jamaica [21], displays a very similar sequence identity to both EuMV-PR (A-strain, 95% identity) and EuMV-Jal (B-strain, 95.4% identity). Therefore, the relationship between EuMV isolates belonging to supposedly distinct strains should be experimentally addressed.

In this work we report the complete molecular characterization of EuMV-Jal, which was found infecting peppers and weeds in Jalisco, Mexico, and was shown to be incompatible in replication with EuMV-YP in reassortment experiments. The genomic analysis of this novel EuMV strain led to the unforeseen discovery of an assemblage of DNA-A homologous sequences in the intergenic region of its DNA-B, whose position and arrangement is conserved in several begomovirus species, hence suggesting the intriguing possibility of a functional role of those atypical sequences in the infective cycle of EuMV and its relatives.

## Results

### Isolation of a new strain of *Euphorbia mosaic virus*

During Autumn 2005, a survey of farming fields infested with whiteflies in the state of Jalisco, Mexico, was undertaken. Pepper plants exhibiting a variety of symptoms (including leaf curling and crumpling, yellow veins, deformed fruits, and stunted growth) were observed in fields of three Jalisco localities. Leaf samples from 63 symptomatic weeds and pepper plants were collected, and total DNA extracts were tested for the presence of begomoviruses using polymerase chain reaction (PCR) with several pairs of degenerated primers (see Methods). More than 80% of the examined samples were PCR-positive and sequence analyses of the amplicons revealed that the majority of the symptomatic plants were infected by begomoviruses belonging to two different species, *Pepper huasteco yellow vein virus *(PHYVV) and *Pepper golden mosaic virus *(PepGMV), which commonly infect pepper and tomato crops throughout the north and central areas of Mexico [22-24]. Partial DNA-A sequences of a third begomovirus were obtained from two pepper samples from the Castillo locality (close to the Pacific coast, coordinates 19°45'00'' N; 104°23'30'' W), one *Nicotiana glauca *plant ("tabaquillo") collected at Sayula (coordinates 19° 47'55'' N; 103°46'05'' W) and one *Euphorbia heterophylla *plant collected at Teocuitatlán (coordinates 20°12'30'' N; 103°30'00'' W). In the four cases the plants were co-infected with either PHYVV or PepGMV. The complete sequence of the DNA-A and DNA-B genomic components of the unidentified begomovirus was obtained from overlapped PCR products derived from one pepper plant co-infected with PHYVV (see Methods). Comparisons with sequences available at the GenBank database using BlastN showed that the third pepper-infecting virus was an isolate of *Euphorbia mosaic virus*, displaying a DNA-A overall sequence identity of 95.4%, 92.8% and 92.1% with EuMV isolates from Jamaica [GenBank: DQ395342], Puerto Rico [GenBank: AF068642] and the Yucatan Peninsula [GenBank: DQ318937], respectively.

### Genome organization of EuMV-Jal

**Figure 1:**
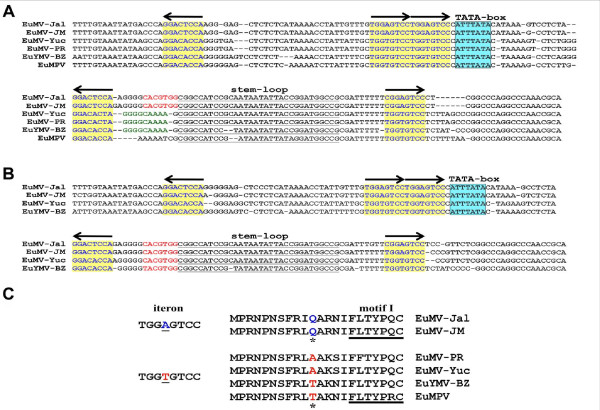
**Comparison of CR sequences from EuMV and relatives**. The alignments of the CR sequences of both (A) DNA-A and (B) DNA-B components from EuMV isolates and related begomoviruses from South America are shown to highlight similarities and differences in relevant *cis*-acting elements. Putative Rep-binding elements (iterons) are shaded in yellow and their relative orientation is depicted by black arrows; the sequence with the potential to form a stem-loop structure is highlighted in black and underlined. The TATA box of the leftward promoter is shaded in blue. The G-box element is shown in red letters, and the "GYA box" conserved in members of the SLCV clade is represented in green letters. (C) Differences in the nucleotide sequence of the iterons and the amino acid sequence of the Rep-IRD of EuMV-Jal and relatives are highlighted. Virus acronyms and GenBank accession numbers are listed in Table 1.

The EuMV-Jal genome exhibited a genetic organization typical of NW-Beg. The DNA-A molecule [GenBank: DQ520942] was 2609 nt in length, and encoded five genes (*cp*, *rep*, *trap*, *ren *and *AC4*). The DNA-B molecule [GenBank: HQ185235] was 2590 nt in size, and contained two major ORFs (*BV1 *and *BC1*). The common region (CR) of EuMV-Jal DNA-A and DNA-B encompassed 169 and 170 nt, respectively, with 98% identity. The CR contained the origin of replication comprising the conserved hairpin element and five iterons (GGAGTCC) that displayed the characteristic arrangement of the viruses belonging to the SLCV-cluster [15,16]. Comparisons of EuMV-Jal CR with the homologous region of other EuMV isolates revealed that EuMV-Jal and EuMV-JM have a DNA-A replication origin with a composition of putative *cis*-acting elements different to the homologous *Ori *of EuMV-YP and EuMV-PR. Indeed, in addition to harbor iterative elements with a distinct nucleotide sequence, the EuMV isolates from Jalisco and Jamaica display a G-box motif in the immediate vicinity of the conserved hairpin element, which is absent in the DNA-A of EuMV-PR and EuMV-YP (Figure 1A). The later viruses display instead a conserved motif (GGGGCAAAA) that is characteristic of most members of the SLCV-clade (our unpublished data). In contrast with the differences observed between the DNA-A components, comparisons of the DNA-B CR revealed a similar modular organization in all EuMV isolates, with a G-box motif adjacent to the hairpin element (Figure 1B). A similar organization of the DNA-B CR is observed in *Euphorbia yellow mosaic virus *(Fernandes et al., unpublished) [GenBank: FJ619507 and FJ619508], a recently described begomovirus from Brazil, that is a distant relative of EuMV (Figure 1B).

### Phylogenetic relationships

**Figure 2:**
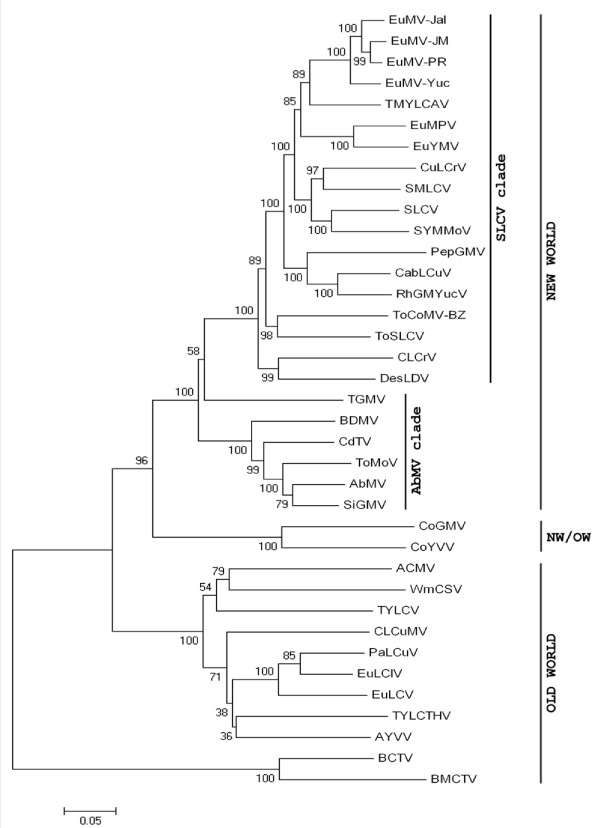
**Phylogenetic relationships of *Euphorbia mosaic virus***. The tree was constructed using Neighbor-joining algorithm implemented by MEGA4 software (66). Branch strengths were evaluated by constructing 1000 trees in bootstrap analysis by step-wise addition at random. Bootstrap values are shown above or under the horizontal line. The vertical distances are arbitrary, whereas the horizontal distances are drawn to scale with the bar indicating 0.05 nucleotide replacements per site. Curtoviruses (*Beet curly top virus *and *Beet mild curly top virus*) were used as out-groups. Virus acronyms and GenBank accession numbers are listed in Table 1.

A phylogenetic tree based on the full-length DNA-A of four EuMV isolates, 20 NW-Beg and several bipartite and monopartite OW-Beg (Table 1), was generated using the neighbor-joining method with 1,000 bootstraps replications (Figure 2). The analysis indicated a close relationship between the EuMV isolates from Mexico and the Caribbean basin with the following three begomoviruses from South America: *Tomato mild yellow leaf curl Aragua virus *(TMYLCAV) from Venezuela [GenBank: AY927277], *Euphorbia mosaic Peru virus *(EuMPV) [25], and *Euphorbia yellow mosaic virus *(EuYMV) from Brazil. This grouping was well-supported by both the phylogenetic analysis (bootstrap value 84) and the pairwise-identity analyses (Table 2), thus defining a sub-lineage within the SLCV clade that is broadly distributed in the American continent. A phylogenetic analysis based on the full-length DNA-B sequences produced similar results for the EuMV subclade and the group of cucurbit-infecting viruses (data not shown), but not for other members of the SLCV lineage that were placed into groups that are not congruent with the phylogeny derived from their DNA-A sequences. The incongruent phylogenies of DNA-A and DNA-B components of some begomoviruses is generally indicative of recombination and/or reassortment events [6,26].

**Table 1:** Names, acronyms, and GenBank accession numbers of the geminiviruses used in this study

Name	Acronym	Accession number	
		DNA-A	DNA-B
Abutilon mosaic virus	AbMV	NC_001928	NC_001929
African cassava mosaic virus	ACMV	NC_001467	NC_001468
Ageratum yellow vein virus	AYVV	NC_004626	
Bean calico mosaic virus	BCaMV	NC_003504	NC_003505
Bean dwarf mosaic virus	BDMV	NC_001931	NC_001930
Bean golden yellow mosaic virus	BGYMV	NC_001439	NC_001438
Beet curly top virus	BCTV	NC_001412	
Beet mild curly top virus	BMCTV	NC_004753	
Cabbage leaf curl virus	CabLCV	NC_003866	NC_003887
Chino del tomate virus	CdTV	NC_003830	NC_003831
Corchorus golden mosaic virus	CoGMV	NC_009644	NC_009646
Corchorus yellow vein virus	CoYVV	NC_006358	NC_006359
Cotton leaf crumple virus	CLCrV	NC_004580	NC_00481
Cotton leaf curl multan virus	CLCuMV	NC_004607	
Cucurbit leaf crumple virus	CuLCrV	NC_002984	NC_002985
Desmodium leaf distortion virus	DeLDV	NC_008494	NC_008495
Euphorbia leaf curl virus	EuLCV	NC_005319	
Euphorbia leaf curl India virus	EuLCIV	EU194914	
Euphorbia mosaic Peru virus	EuMPV	AM886131	
Euphorbia mosaic virus-Jalisco	EuMV-Jal	DQ520942	HQ185235
Euphorbia mosaic virus-Jamaica	EuMV-JM	FJ407052	EU740969
Euphorbia mosaic virus-Puerto Rico	EuMV-PR	AF068642	
Euphorbia mosaic virus- Yucatan	EuMV-YP	NC_008304	NC_008305
Euphorbia yellow mosaic virus	EuYMV	NC_012553	NC_012554
Papaya leaf curl virus	PaLCuV	AJ436992	
Pepper golden mosaic virus	PepGMV	NC_004101	NC_004096
Pepper huasteco yellow vein virus	PHYVV	NC_001359	NC_001369
Rhynchosia golden mosaic Yucatan virus	RhGMYucV	NC_012481	NC_012482
Sida golden mosaic virus	SiGMV	NC_002046	NC_002047
Squash leaf curl virus	SLCV	NC_001936	NC_001937
Squash mild leaf curl virus	SMLCV	NC_004645	NC_004646
Squash yellow mild mottle virus	SYMMoV	NC_003865	NC_003860
Tomato common mosaic virus-Brazil	ToCoMV-BZ	NC_010835	NC_010836
Tomato golden mosaic virus	TGMV	NC_001507	NC_001508
Tomato mild yellow leaf curl Aragua virus	TMYLCAV	NC_009490	NC_009491
Tomato mottle virus	ToMoV	NC_001938	NC_001939
Tomato severe leaf curl virus	ToSLCV	DQ347947	
Tomato yellow leaf curl Thailand virus	TYLCTHV	X63015	X63016
Tomato yellow leaf curl virus	TYLCV	X15656	
Watermelon chlorotic stunt virus	WmCSV	NC_003708	NC_003709

**Table 2:** Percentages of sequence identities between EuMV-Jal and selected begomoviruses (DNA and predicted proteins*)

	DNA-A	IR-A	CP*	AC1*	AC2*	AC3*	AC4*	DNA-B	IR-B	BV1*	BC1*
Virus											
**ACMV**	45	25	66	49	43	42	19	27	22	24	41
**BCaMV**	76	50	92	86	78	77	64	55	28	73	83
**BGYMV**	64	37	91	63	70	78	11	48	22	67	80
**CdTV**	67	43	92	63	67	78	30	51	27	71	78
**CoYVV**	51	24	87	43	51	43	19	41	22	52	71
**CuLCrV**	77	46	91	83	71	71	72	51	27	66	76
**DesLDV**	72	44	91	80	66	73	58	50	23	64	77
**EuMPV**	77	52	93	86	81	76	58	-	-	-	-
**EuYMV**	77	51	90	85	80	76	62	52	35	73	82
**EuMV-JM**	**95**	**91**	98	**97**	**97**	**95**	88	**86**	**73**	**96**	**98**
**EuMV-PR**	92	82	**99**	96	93	91	**91**	-	-	-	-
**EuMV-YP**	92	80	99	93	93	91	87	85	63	94	**98**
**PepGMV**	72	50	90	80	71	75	14	48	25	64	74
**PHYVV**	59	33	89	49	50	63	12	47	25	66	74
**RhGMYV**	76	54	94	86	70	70	66	51	31	69	78
**SLCV**	78	57	94	82	72	80	77	50	30	63	80
**ToCoMV-BZ**	73	43	90	85	64	72	57	52	31	63	77
**TMYLCAV**	84	66	95	88	87	80	82	56	43	75	83
**TYLCTHV**	48	28	68	48	43	39	22	25	19	21	39

### Recombination analysis

The differences between the strains A and B of EuMV regarding nucleotide sequence and modular organization of the *Ori *region could be indicative of either divergent molecular evolution or intermolecular recombination between co-infecting begomoviruses [27,28]. To search for potential recombinant sequences in the genome of EuMV strains, we analyzed sequence alignments that included the DNA-A of the four EuMV isolates under exam, as well as diverse sets of begomoviruses of the SLCV clade, using the suite of programs for detection of recombinant breakpoints integrated within the RDP package [29]. The analysis identified a ~210-nt long EuMV genomic region (recombinant breakpoints at positions 2432 and 33 of EuMV-Jal DNA-A) as a fragment of possible recombinant origin, which includes the entire common region (~ 170-nt) as well as the first 44 nucleotides of the *rep *gene, encompassing the IRD-coding sequence [17]. The plausible recombinant origin of this DNA fragment is underscore by direct comparisons of the DNA-A components from EuMV-JM and EuMV-PR, which are members from different strains exhibiting very high sequence identity (97.4%) along a segment encompassing ~2,400 out the 2,609-nt of its DNA-A, a fact that is in clear contrast with the low sequence identity (77.5%) displayed in the 210-nt genomic region flanked by the recombinant breakpoints detected by our analysis.

The assembled data suggest that EuMV A-strain viruses are the product of an intermolecular recombination event involving an EuMV-JM-related virus (the major parent) and a virus closely related to *Calopogonium golden mosaic virus *(CpGMV) [GenBank: AF439402] which might have donated the ~210-nt fragment with the viral replication module. This DNA segment, which is entirely identical in sequence between EuMV-PR and EuMV-YP, is shared with CpGMV at 90% of nucleotide identity. Two additional observations support the hypothesis of intermolecular recombination: (1) The absence of a G-box element within the CR of the DNA-A component of EuMV-YP, that is nevertheless present in their cognate DNA-B component (see Figure 1); and (2) The lower than expected sequence identity of the EuMV-YP common region (i.e., 86%) that is in contrast with the high identity of the CR of both EuMV-Jal and EuMV-JM (98% and 96%, respectively) [20,21].

### Experimental infection of host plants

**Figure 3:**
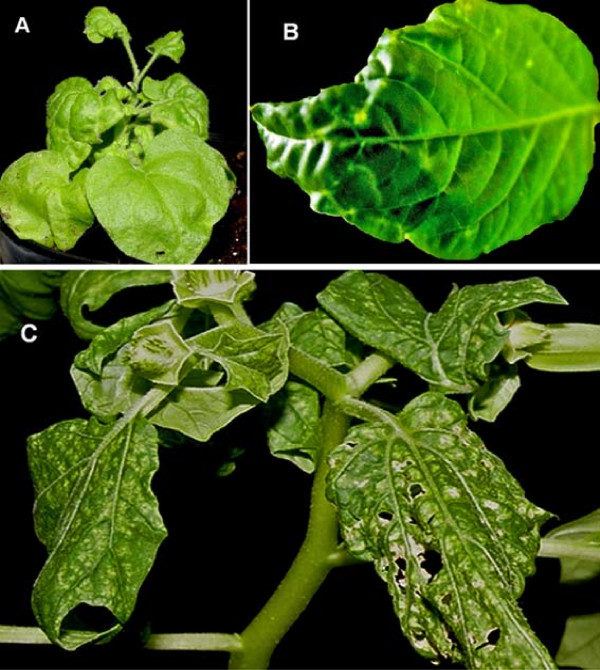
**Symptoms induced by EuMV-Jal in experimentally infected plants**. (A) *Nicotiana benthamiana*, (B) *Capsicum annum*, and (C) *Datura stramonium*.

EuMV-Jal was identified in four field samples that contained an additional, distinct begomovirus, as mentioned above. In order to examine experimentally EuMV-Jal in single plant infections, we generated infectious clones of both DNA-A and DNA-B components (see Methods), and carried out biolistic inoculation of these clones into four plant species: *Datura stramonium*, *Nicotiana benthamiana*, pepper (*Capsicum annum*), and zucchini (*Cucurbita pepo*). All solanaceous species were susceptible and developed systemic symptoms at 10-12 dpi, while the zucchini plants did not show symptoms and no viral DNA was detected by PCR in their tissues at 14 dpi. Symptoms of EuMV-Jal infection varied between plant species. In *N. benthamiana *the symptoms included leaf crumpling, greenish mosaics and shortened internodes (Figure 3A). In pepper plants the first symptom was the appearance of small green spots that progressed into a pale green mosaic and moderate downward leaf curling; a few small necrotic spots were also observed in several plants (Figure 3B). The most severe symptoms were observed in *D. stramonium *plants, whose leaves showed deformation and extensive green and yellow mottle covering most of the foliar surface, progressing in time to necrotic lesions leading to the destruction of significant parts of the foliar lamina (Figure 3C). In all, the symptoms induced by EuMV-Jal in the examined three plant species were very similar to those generated by infection of EuMV-YP [20], hence suggesting that these viruses express equivalent pathogenesis factors, as expected from the high amino acid sequence identity of their predicted proteins (Table 2).

### EuMV-Jal and EuMV-YP are incompatible in replication

The replication modules of the EuMV strains A and B exhibit two main differences: 1) their iterons display a different nucleotide N within the GGNGTCC core, and 2) the iteron-related domain of their Rep proteins have a different amino acid residue at position X_3 _of the IRD core FX_1_L*X_3 _[17], that is either FRLA or FRLT in A-strain viruses, and FRLQ in B-strain members (Figure 1C). These observations suggest the intriguing possibility that EuMV strains A and B could be incompatible in replication. To answer this question we carried out reassortment experiments with the EuMV-Jal and EuMV-YP genomic components. The four possible combinations A+B of the cloned viral DNAs were biolistically inoculated into *N*. *benthamiana *plants, that were subsequently scored for the appearance of disease signs.

**Figure 4:**
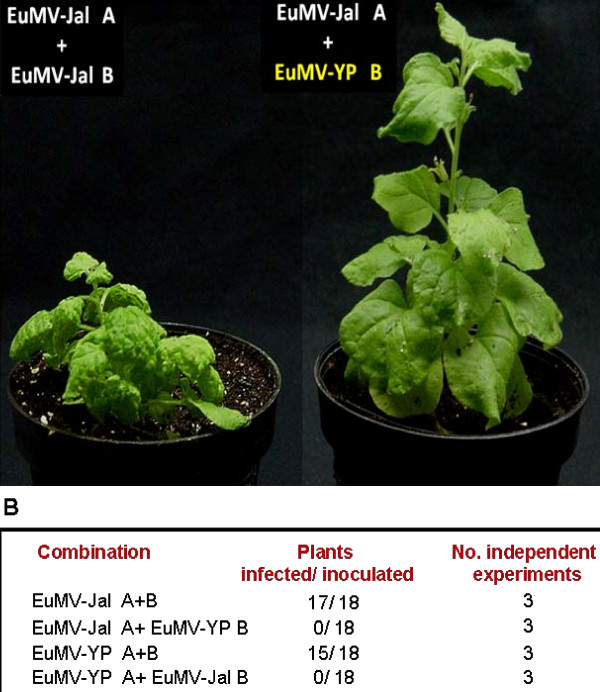
**EuMV-Jal does not form viable reassortants with EuMV-YP**. (A) *N*. *benthamiana *plants inoculated with either the two genomic components of EuMV-Jal (left), or the heterologous combination EuMV-Jal DNA-A/EuMV-YP DNA-B (right). Plants were inoculated by microparticle bombardment with 5 μg of each DNA component, and photographed 26 days after inoculation. (Panel B) Results of the reassortment experiments between EuMV-YP and EuMV-Jal. Negative controls (plants inoculated with the empty vector) were included in the three independent experiments but the data were omitted for simplicity.

Systemic symptoms developed at 10-12 dpi in most plants inoculated with the homologous mixtures (i.e., EuMV-Jal [A+B], and EuMV-YP [A+B]); in contrast, the plants bombarded with the heterologous combinations (i.e., EuMV-Jal [A]/-YP [B] and its reciprocal, EuMV-YP [A]/-Jal [B]) displayed no symptoms at 12 dpi, and remained symptomless until the end of the experiment, at 30 dpi (Figure 4A). These experiments were repeated three times, six plants for each combination, with similar results obtained (data in Figure 4B). All plants inoculated with cognate viral components scored positive for presence of both EuMV DNA-A and DNA-B, based on PCR detection of a ~1300-bp fragment encompassing a part of the *rep *and *cp *genes and the entire DNA-A intergenic region, and a ~1400-bp segment comprising the DNA-B IR and a part of both *BV1 *and *BC1 *genes. In contrast, none of the newly emerged leaves of plants bombarded with the heterologous combinations of EuMV genomic components tested positive for presence of EuMV DNA-B, although a few plants (5 out 36) were PCR-positive for DNA-A at 14 dpi, but not at 28 dpi (data not shown). These results indicate that viral factors required for replication are not exchangeable between EuMV-Jal and EuMV-YP.

### EuMV BV1 promoter contains a short sequence homologous to Rep gene

**Figure 5:**
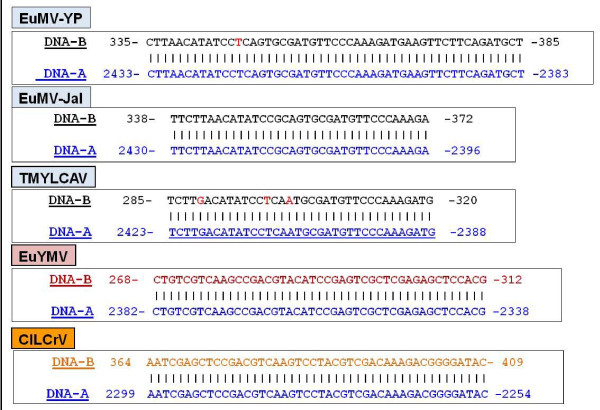
**Nucleotide sequence of s*Rep*HS elements**. The upper sequence correspond to the DNA-B and the lower one to the cognate DNA-A. Letters in red within the s*Rep*HS elements of EuMV-YP and TMYLCAV denote differences with the homologous sequence of EuMV-Jal._- _Virus acronyms are listed in Table 1.

In the course of a meticulous scrutiny of the DNA-B intergenic region of EuMV-Jal to identify potential *cis*-regulatory elements involved in the transcriptional control of the *BC1 *and *BV1 *genes, we unexpectedly discovered a 35-bp DNA stretch displaying 100% sequence identity with a segment of the homologous *rep *gene. This sequence is located ~150-nt upstream to the *BV1 *gene (nucleotides 337-372) and contains the coding information for aa residues 15 to 25 of EuMV-Jal Rep (i.e., FLTYPQCDVPK) that includes the conserved Motif I of the RCR initiators [30]. No additional sequences homologous to the *rep *gene were found in the *BV1 *promoter region. The finding of a short sequence apparently derived from the cognate DNA-A within the noncoding region of EuMV-Jal DNA-B was intriguing and prompted further scrutiny of other EuMV DNA-B components. In all the examined cases a short *Rep *homologous sequence (s*Rep*HS) was found within the *BV1 *promoter region, which in EuMV-JM is similar to the EuMV-Jal element in both sequence and length (35-nt), but that is longer in EuMV-YP that displays a DNA stretch 51-nt in length identical to a segment of its cognate *rep *gene (Figure 5). A search for analogous elements in the DNA-B IR from all members of the SLCV clade revealed that s*Rep*HS elements are not common, being identified only in two close relatives of EuMV, namely, TMYLCAV from Venezuela and EuYMV from Brazil. The TMYLCAV s*Rep*HS element is similar but not identical in both length (36-nt) and nucleotide sequence (88% identity) to the equivalent sequence of EuMV-Jal (Figure 5). In contrast, the s*Rep*HS identified in EuYMV DNA-B is different in both length (45-nt) and nucleotide sequence (< 30% identity) to the analogous elements of EuMV strains. Indeed, the EuYMV s*Rep*HS element corresponds to a distinct segment of the cognate *rep *gene, encoding the Rep aa residues 40-53 (i.e., VVKPTYIRVARELH) instead of Rep residues 15-25 encoded by the s*Rep*HS elements of TMYLCAV and EuMV. Notwithstanding its divergent nucleotide sequence, the EuYMV s*Rep*HS element is 100% identical in nucleotide sequence to a segment of its cognate *rep *gene, like in EuMV and TMYLCAV (Figure 5) and is located at a position equivalent to the s*Rep*HS in the latter viruses.

### s*Rep*HS upstream sequences are similar to CP promoters

The conservation of s*Rep*HS elements in the DNA-B intergenic region of EuMV and their relatives suggests that those atypical sequences might play a defined role in the infective cycle of these viruses. Since the s*Rep*HS elements do not contain a start codon and are not a part of a distinctive ORF, it seems plausible that its function, if any, involves an intermediary RNA molecule. This notion naturally led us to suggest the existence of a functional promoter next to the s*Rep*HS element.

**Figure 6:**
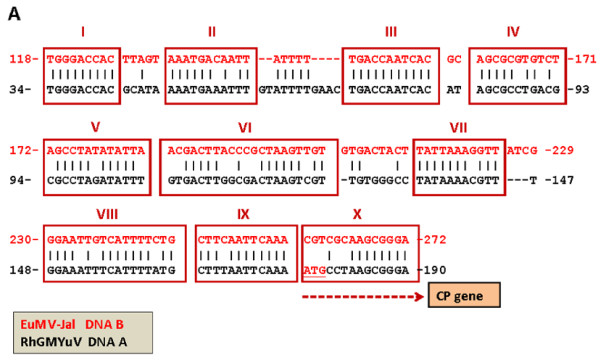
**Potential internal promoter in the DNA-B intergenic region of EuMV**. Alignment of a domain of the DNA-B intergenic region of EuMV-Jal with the coat protein (*CP*) gene promoter of *Rhynchosia golden mosaic Yucatan virus *[GenBank: GQ352453]. The blocks of high sequence similarity encompassing more than eight nucleotides are "phylogenetic footprintings" (PFp; boxed). Putative *cis*-acting elements within the PFp (identified by roman numerals) are as follows: I) Conserved Late Element, CLE (inverted); II) salycilic acid responsive element (inverted, restricted similarity); III) A pathogen-elicitor responsive element (Box W1) overlapped with a CCAAT box; IV) conserved element with indeterminated function; V) sequence highly similar to the 3'end of the TGMV CP promoter "region C", that functions as transcriptional negative element [67]; VI) potential auxin-responsive element (ARE, at the block 3'end); VII) TATA box; VIII) canonical salycilic acid responsive element; IX) ethylene-responsive element (ERE)-like motif. The block "X" includes the first segment of the RhGMYuV *CP *gene. Notice the 8-nt long stretch of identical sequence between the non-coding sequence of EuMV-Jal DNA-B and the coding sequence of RhGMYuV.

In order to identify potential IR internal promoters, we analyzed the sequences upstream to s*Rep*HS in all members of the EuMV lineage using a phylogenetic-structural approach. This methodology entails the identification of "phylogenetic footprintings" (i.e., putative binding sites for transcription factors) and conserved arrays of them, named "Conserved Modular Arrangements" (CMAs), in non-coding regions of evolutionarily-related DNA sequences [31,32]. The new analysis exposed a DNA-B IR domain ~160-bp-long exhibiting a remarkable similarity both in overall nucleotide sequence and modular organization, to *CP *promoters of viruses that belong to the SLCV clade. The example showed in Figure 6 illustrates the remarkable similarity between the *CP *promoter-like (*CP*prom-L) domain of EuMV-Jal IR and a 156-nt segment of the *CP *promoter of *Rhynchosia golden mosaic Yucatan virus *(RhGMYuV), a recently described virus of the SLCV lineage [33]. The similarity between these DNA-B and DNA-A sequences, respectively, includes nine phylogenetic footprintings in a definite order, and it is extended beyond the start codon of RhGMYuV *cp *gene including a block of 8-nt of coding sequence that is conserved in the non-coding sequence of EuMV-Jal DNA-B.

The demarcated *CP*prom-L domain of the DNA-B IR includes several putative *cis*-regulatory elements that were identified by consulting plant transcription factors databases like PlantCare [34] and PLACE [35]. Among the identified potential *cis*-acting motifs there were well-characterized regulatory elements such as the "Conserved Late Element" (CLE) [36], the CCAAT box, and several elements that confer responsiveness to a variety of plant hormones (see Figure 6 legend). Among these sequences there is a 12-bp long element (consensus: CTTTAATTCAAA) which is identical to a conserved sequence immediately adjacent to the *cp *gene in more than 75% of the known begomoviruses from America (Cardenas-Conejo et al., unpublished data). The AATTCAAA motif of the former element is both a putative ethylene-responsive element (ERE) and a binding-site for nuclear factors of carnation, tomato and *Solanum melongena *[37-39]. In addition, this motif constitutes the 8-nt long leader sequence of the *CP*mRNA of *Tomato golden mosaic virus *(TGMV) [40]. The ERE-like motif is located downstream to the actual TATA-box of NW-Beg *CP *promoters, at a similar distance (21-29 bp) to that observed between the ERE and a putative TATA box in the *CP*prom-L domain [Additional file 1: Supplemental Figure S1a]. Taken as a whole, these remarkable similarities between noncoding DNA regions from two different genome components of separate begomovirus species, can hardly be explained by random sequence convergence; rather, they strongly suggest that the DNA-B *CP*prom-L domain of EuMV and relatives is evolutionarily derived from a begomovirus *CP *promoter.

### Distantly related begomoviruses contain s*Rep*HS elements

The existence of s*Rep*HS elements in the DNA-B IR of viruses belonging to a minor lineage of the SLCV clade is an interesting evolutionary enigma. To determine whether analogous elements actually exist in other viral lineages, we searched for *rep *homologous sequences in the DNA-B IR of begomoviruses belonging to 12 major and minor clades, distributed in several continents. The analysis of ~60 members of those lineages led us to the identification of only two additional begomoviruses displaying s*Rep*HS in the *BV1 *upstream region: TGMV and the recently described *Cleome leaf crumple virus *(ClLCrV) [41]. These viruses are native from Brazil, like EuYMV, but do not belong to the SLCV clade. The s*Rep*HS element of ClLCrV is 100% identical to a 46-nt-long segment of its cognate *rep *gene, encoding the aa residues 97 to 110 (SSSDVKSYVDKDGD), that comprise the conserved RCR Motif 3 (underlined) [30]. On the other hand, the TGMV s*Rep*HS element is only 88% identical to a 52-nt-long segment of its cognate *rep *gene, encoding the aa residues 255 -271 (NKVEYNVIDDVTPQYLK) of this replication initiator, that include the Walker B-motif (underlined), a critical aa sequence of the protein ATPase/helicase domain [42,43].

The upstream sequences of TGMV and ClLCrV s*Rep*HS elements were examined, but no significant similarity between them nor with the *BV1 *promoter region of EuMV lineage viruses was found. However, a careful re-examination of sequences nearby to the 5'end of ClLCrV s*Rep*HS revealed a 23-bp sequence with partial dyad symmetry that is well-conserved both in sequence and in position relative to the s*Rep*HS element in all viruses of the EuMV cluster [Additional file 1: Suppl. Figure S1b]. The consensus of this conserved sequence includes a palindromic core with the repeated motif TTGTGGTCC, similar to the CLE, a functional target of plant transcriptional activators [44,45] that has been involved in TrAP-mediated activation of the CP promoter in some begomoviruses [36]. No sequence similar to the latter symmetric element was found in the *BV1 *promoter region of TGMV. In fact, the s*Rep*HS of the latter virus differs from the analogous elements in ClLCrV and the EuMV subclade viruses in several other important features: (1) It is not 100% identical to the corresponding segment of its cognate *rep *gene; (2) It has opposite polarity compared to all other known s*Rep*HS elements; (3) It is closely located downstream to a putative internal promoter that does not exhibit significant similitude with *CP *promoters of SLCV clade viruses (data not shown). It is relevant to point out here that TGMV and ClCrV are grouped, on the basis of their full-length DNA-A sequences, within the Brazilian cluster of NW-Beg [41], but they have very divergent DNA-B components. Thus, our finding of the s*Rep*HS-associated semi-palindromic sequence in ClLCrV DNA-B suggests an actual relationship of the latter with the homologous genomic components of EuMV and relatives, a notion that is supported by a recent study that groups the ClLCrV DNA-B with viruses of the EuMV lineage [41].

## Discussion

In this study, we described the molecular and biological characterization of a novel strain of *Euphorbia mosaic virus *that was isolated from pepper plants in the state of Jalisco, Mexico, near to the Pacific shoreline. This virus displays 92% sequence identity with EuMV-YP, that was isolated in the same country but in a distant region, close to the Atlantic coastline [20]. These viruses differ in two important features of their DNA-A replication origin region: the nucleotide sequence of their iterons, and the presence or absence of a G-box element, a *cis*-acting sequence which is critical for *Rep *promoter activity in some NW-Beg [46]. The differences observed in the predicted Rep-binding sites of EuMV-Jal and EuMV-YP prompted us to explore experimentally their ability to form viable reassortants in pseudorecombination tests. The results of these experiments confirmed the presumption of replication incompatibility between EuMV-YP and EuMV-Jal, thus demonstrating that the latter is a new, biologically-defined strain exhibiting different replication specificity.

The finding of begomovirus strains that are not able to form viable reassortants is somehow bewildering because the common definition of a virus species is "A...class of viruses that constitutes a replicating lineage and occupies a particular ecological niche." [47,48]. Accordingly, it is not expected that strains of a virus species would be incompatible in replication because that implies that they do not constitute an actual replicating lineage. Nonetheless, it is generally recognized that several strains of begomoviruses probably are not complementary in replication because they display different putative *cis*- and *trans*-acting replication specificity determinants [7,17]. There is at least one report of strains belonging to a bipartite begomovirus that are not equivalent in replication functions (the "severe" and "mild" strains of *Tomato leaf curl New Delhi virus*, ToLCNDV) [49]. However, that case is different from the one examined here because the "mild" phenotype of one ToLCNDV strain seems to be related to an inefficient *trans*-replication of the "cognate" DNA-B, which displays Rep binding-sites different to those of the associated DNA-A [49,50].

The case of the EuMV strains is significant because it is paradigmatic of an apparently common theme in begomovirus evolution, i.e., the sudden change of virus replication specificity determinants by intermolecular recombination between co-infecting viruses [27,51]. Indeed, the recombination analysis of EuMV isolates indicates that viruses of the EuMV A-strain probably evolved by an event of DNA intermolecular exchange involving a member of the EuMV B-strain and a virus related to CpGMV, which had donated a ~210-bp DNA segment encompassing the region of the virus replication origin and the first 44 nucleotides of the *rep *gene. If this hypothetical scenario is accurate, then the recombination event should have changed simultaneously both the iterons and the Rep aa residues interacting with them, thus maintaining the proper matching of *cis*- and *trans*-acting replication determinants in the recombinant DNA-A component.

Diverse studies have identified the sequences encompassing the viral strand replication origin and the *rep *gene segment encoding the Rep N-terminal domain, as the regions of geminivirus genomes most frequently exchanged during recombination [28,51-53]. This is consistent with the known genome localization of the Rep-binding sites and the coding sequence of the Rep domain that contains the putative DNA-binding specificity determinants of this protein, which have been theoretically mapped into the first 75 aa residues [17,54]. Consequently, a recombination event involving a genome portion as small as 200 to 360-bp might confers a completely different replication phenotype to begomoviruses involved in mixed infections, as presumably is the case for the EuMV strains.

Since that intermolecular recombination is/has been a major force in the evolution of geminiviruses, the concepts of both "species" and "strains" should be adapted to the peculiar nature of these entities, that are genetic mosaics in continual change, different in quality to cellular organisms. In fact, it is altogether possible that a significant part of the currently recognized begomovirus species would not constitute "replicating lineages" in a strict sense, as would be the case of EuMV, according to our experimental data. For instance, a thorough sequence analysis entailing the identification of the putative *cis*- and *trans*-acting Replication Specificity Determinants (RSDs) of the 182 recognized begomovirus species summarized by Fauquet et al. in 2008 [7] revealed the existence of 34 species that include at least two groups of viruses exhibiting distinct putative RSDs, analogous to the strains A and B of EuMV. Furthermore, some ICTV-accepted species as *Ageratum yellow vein Hualian virus*, *Honeysuckle yellow vein virus*, *Tomato leaf curl Bangalore virus*, *Tomato leaf curl Philippines virus, Tomato leaf curl Taiwan virus*, and ToLCNDV, include three classes of viruses differing in their putative RSDs, and one viral species, *Ageratum yellow vein virus*, comprises four types of viruses harboring distinct replication modules, plausibly acquired through independent episodes of intermolecular recombination (Arguello-Astorga, unpublished data). In view of the significant number of begomovirus species with variants that are seemingly analogous to the strains of EuMV, it would be important to establish a formal distinction between strains with similar RSDs, that represent actual replicating lineages, and replication-incompatible strains, that apparently do not.

### What is the function of the DNA-B s*Rep*HS elements?

During the analysis of the intergenic region of EuMV-Jal DNA-B we discovered a short DNA stretch identical to a segment of the *rep *gene coded in the cognate DNA-A. It was subsequently find out that analogous s*Rep*HS elements exist in the DNA-B IR of at least five begomovirus species, all them from the New World: EuMV from Mexico and the Caribbean basin, TMYLCAV from Venezuela, and EuYMV, ClLCrV and TGMV from Brazil. With the exception of the short *rep *homologous sequence in the DNA-B IR of TGMV (that seems to be evolutionarily unrelated) the s*Rep*HS elements of begomoviruses have in common several characteristics. All of them: (1) are short sequences, ranging from 35 to 51 nucleotides in length; (2) are 100% identical in nucleotide sequence to a segment of its cognate *rep *gene; (3) have opposite polarity than the *rep *gene; (4) are located 65 to 80-nt downstream to a putative internal promoter highly similar to *CP *promoters of viruses of the SLCV clade (ClLCrV being an exception); (5) are positioned 7-9 nt downstream to a 23-bp partly palindromic element with a repeated motif similar to the CLE; and (6) are situated 115 to 145-nt upstream to the *BV1 *gene. In contrast, the s*Rep*HS elements of viruses that are distantly related, like EuMV, EuYMV and ClLCrV, have entirely different nucleotide sequences (see Figure 5), because the coding sequence represented in those elements corresponds to distinct sections of the cognate *rep *gene.

An intriguing observation is that the identified s*Rep*HS elements reproduce sequences encoding conserved aa motifs which are critical for Rep functions. For example, the s*Rep*HS of EuMV strains and TMYLCAV correspond to the coding sequence of RCR Motif 1; the equivalent element of ClLCrV encodes the RCR Motif 3, and the analogous s*Rep*HS of TGMV duplicate the *rep *sequence encoding the Walker B motif of ATPases/helicases. An apparent exception is the s*Rep*HS of EuYMV, which displays the coding sequence of a conserved Rep motif of unknown function. The evolutionary conservation of s*Rep*HS elements and the associated sequence motifs, suggests that those atypical elements play a definite but hitherto unknown function in the viral infective cycle. In absence of any factual data it is only feasible to speculate about the possible function(s) of the s*Rep*HS on the basis of their common characteristics.

Certainly, the most remarkable feature of the s*Rep*HS elements is its complete identity in nucleotide sequence with a specific segment of the *rep *gene in the cognate DNA-A component, because the evolutionary preservation of such an absolute matching between specific segments of distinct, physically separated DNA molecules, should involve very strong selective pressures against mutations diminishing the identity between the former DNA sequences. Therefore, the function of the s*Rep*HS elements is most likely related to a process that requires a perfect or very high complementarity between DNA and/or RNA molecules, such as the gene regulation by microRNAs (miRNAs).

The miRNAs are ~22-nt-long noncoding RNAs that posttranscriptionally regulate gene expression by binding to specific mRNAs, thus repressing its translation and/or inducing its degradation [55]. Several DNA viruses (i.e., herpesviruses, adenoviruses, ascoviruses and polyomaviruses) encode miRNAs which participate in the regulation of some processes of the viral infection cycle [56,57]. For example, the simian virus 40 (SV40) encodes a single miRNA which lie antisense to the viral mRNA encoding the T-antigen, a multifunctional protein essential for virus replication. This miRNA is expressed late in infection, hence promoting the T-antigen mRNA degradation and downregulating the synthesis of this protein at late stages of the SV40 replication cycle [58]. In close analogy with SV40 miRNA, the s*Rep*HS elements of begomoviruses are single, discrete noncoding DNA sequences highly similar to a specific segment of the gene encoding the viral replication protein. Further analogies between those heterologous viral sequences are the following: (1) The genomic location of the miRNA, but not its nucleotide sequence, is conserved among polyomaviruses (i.e., SV40, Merkel cell virus, human BK virus, JC virus, and mouse polyomavirus) [59-61]; similarly, the location of s*Rep*HS elements within the DNA-B intergenic region, but not its specific sequence, is conserved among begomoviruses (data from this study); (2) The temporal expression of the SV40 miRNA, that is restricted to the late stage of infection, is similar among all the examined polyomaviruses [57,59]; likewise, although the temporal expression of begomovirus transcripts including the s*Rep*HS region (if any) is unknown, it is plausible than them would be late expressed, because the hypothetical promoter that lead its transcription is similar to begomovirus *CP *promoters, which are typically active at the late phase of the viral infection cycle [1,36]; (3) Like the polyomavirus pre-miRNAs, the DNA-B sequences encompassing s*Rep*HS and the neighboring sequences, have the potential to form extensive hairpin structures susceptible to cleavage by RNase III enzymes (i.e., Drosha and Dicer) involved in the processing of pre-miRNAs (data not shown). Taken together, these lines of indirect evidence suggest a potential function of the s*Rep*HS elements in the posttranscriptional regulation of Rep expression, a hypothesis that must be experimentally examined.

## Conclusions

The evidence gathered in this study indicates that EuMV-YP and EuMV-Jal, which are members from the strains A and B of *Euphorbia mosaic virus *respectively, are actually incompatible in replication, hence implying that these viruses probably represent distinct replicating lineages in natural ecosystems. The scenario we propose for the origin of the EuMV A-strain viruses involves a recombination event that substituted the DNA-A core replication module of an EuMV B- strain virus, with the analogous genomic region of a virus related to CpGMV. This intermolecular exchange suddenly changed the replication specificity of the recombinant DNA-A, thus triggering the process that led to the evolutionary differentiation of EuMV into two distinct strains. The fact that more than 30 recognized begomovirus species include two or more classes of viruses with distinct putative RSDs (i.e., analogous to the EuMV strains) suggests that intermolecular recombination events that involve the virion-strand origin of replication and the first part of the *rep *gene, are quite common in this group of ssDNA viruses, as has been previously pointed out (51, 52, 53). Another relevant result from this study is the discovery of atypical sequences within the intergenic region of the DNA-B component from some NW-begomoviruses, mostly related to EuMV. These sequences include short fragments of the cognate *Rep *gene located downstream from a potential internal promoter very similar in modular organization to *CP *promoters of viruses of the SLCV clade. Even though we do not know the actual function of these s*Rep*HS elements, several lines of indirect evidence suggest their participation in the posttranscriptional regulation of Rep expression, an intriguing possibility that is currently being examined in our laboratory.

## Methods

### Plant samples and DNA extraction

Samples of symptomatic plants exhibiting leaf curling, yellow or golden mosaic, vein chlorosis and/or stunted growth were collected in several farm fields in the State of Jalisco, Mexico, during 2005. Young leaves from symptomatic pepper plants, as well as a variety of weed plants found as underbrush within the field were gathered. Total nucleic acids were extracted of field samples using a modified version of the Dellaporta method [62].

### PCR-based detection of begomoviruses

Total DNA extracts from 63 symptomatic plants were used as templates in PCR reactions with degenerate primers designed to amplify two overlapping genomic segments encompassing either the complete DNA-A or DNA-B of begomoviruses belonging to the SLCV clade [63]. The primers SL2150-for (GACGGCRTTGGYGTCTTTGGC) and cpYMAC-rev (TTWGASGCATGNGTACATGCCA) were used to amplify a DNA-A segment encompassing the intergenic region and part of both *Rep *and *CP *genes, whereas the primers CP70-for (GGTTGTGAAGGNCCNTGTAAGGTYCA) and SL2150-rev (GCWGCAAAGACACCAAYGCCGT) were utilized to amplify a complementary and partially overlapped DNA-A segment. Amplification of DNA-B sequences was performed with degenerated primers BC1-290-for (GAARTAGTGGAGATCTATGTTRCAYCT) and BV1- 470-rev (CCATGRCTRTGRATYCTWGCRCC), designed to amplify the complete intergenic region together with a part of both the BC1 and BV1 genes. To amplify the remaining part of DNA-B the degenerate primers BC1- 290-rev (CCSATMAGRTGYAACATAGATCTCC) and BV1-310-for (AGGWACRGTNAARATYGARCGTGT) were used. The PCR-products were cloned into the pGEM-T easy vector (Promega) and subjected to Restriction Fragment Length Polymorphism (RFLP)-analysis by using *Eco*R I in combination with either *Hinf *I or *Msp *I. The produced DNA molecules were fractioned in 2.5% agarose gels, and PCR clones with different restriction patterns were sequenced.

### Generation of infectious EuMV clones

To clone the full-length genomic components A and B of EuMV-Jal, the DNA extract of one pepper plant infected with both EuMV-Jal and PHYVV collected at the Castillo locality (see Results) was subjected to rolling circle amplification (RCA) using the TempliPhi kit (GE Healthcare, USA) according to the manufacturer's instructions. The full-length EuMV DNA-A was obtained by cutting the RCA-amplified DNA with Xho I and subsequent cloning of the 2.6 Kb DNA molecule into a plasmid vector. The full-length EuMV DNA-B could not be obtained by a similar procedure after several attempts. Consequently, abutted divergent primers designed over the unique BamHI site in the DNA-B of EuMV-Jal were used in a standard PCR procedure, and the generated 2.6 Kb amplicon was cloned into a pGEM-Teasy vector (Promega). The infectious clones of EuMV-Jal A and EuMV-Jal B were generated as follows: the 0.8-Kb BamHI- XbaI fragment of EuMV-A containing the origin of replication was cloned into the BamHI- XbaI sites of a modified pBlueScript plasmid to create pEu-oriA. Subsequently, a full-length DNA-A of EuMV digested with XbaI was inserted into the XbaI site of pEu-oriA to generate the viral replicon pEuMV1.33A. The infectious clone of EuMV-B was generated by an analogous procedure: the full-length DNA-B cloned into pGEM-T easy was digested with NcoI and re-ligated. This procedure deleted a portion of the viral genome, leaving intact all elements important for replication (1.3-kb), thus generating the pEu-oriB plasmid. Finally, a full-length DNA-B digested with BamHI was cloned into the BamH1 site of pEu-oriB, yielding the infectious clone pEuMV1.5B.

### Plant infection assays

*Nicotiana benthamiana, Capsicum annuum *and *Datura stramonium *plants were inoculated using a low-pressure biolistic method [64]. The target leaves (third- to four-leaf stage) were either directly shot at 100 to 120 psi helium pressure with tungsten particles (0.7 mm, BioRad, Hercules, CA) covered with the EuMV-A and EuMV-B viral DNAs (5 μg), or inoculated mechanically by using carborundum according to a procedure recently described [65]. The inoculated plants were maintained in an insect-free growth chamber (27°C, daily cycle of 16 h light -8 h dark), and subsequently scored for the appearance of disease symptoms. The infection status of the inoculated plants was assessed by visual inspection of symptoms and by PCR analysis of all plants at the end of the experiment.

### Reassortment experiments

Pseudorecombination experiments were carried out by biolistically inoculating seedlings of *N*. *benthamiana *plants with all possible pair combinations of A and B component clones of both EuMV-Jal and EuMV-YP. Infectious clones of EuMV-Jal were partial tandem repeats of either DNA-A or DNA-B components, as mentioned above, whereas in the case of EuMV-YP, cloned monomeric components were used as previously described [20]. A total of 18 seedlings (three independent experiments, six plants each replicate) were inoculated with each one of the four possible combinations of EuMV-Jal and EuMV-YP genomic components. Mock-inoculated negative controls were included for each replicate. The inoculated plants were maintained in a growth chamber (27°C, daily cycle of 16 h light -8 h dark) and scored for the appearance and development of disease symptoms during 4-5 weeks. All plants, both symptomless and symptomatic, were tested for the presence of viral DNA in newly emerged leaves at 14 dpi by PCR-based detection, using both DNA-A and DNA-B specific primers. Asymptomatic plants were re-examined by PCR at 28 dpi, to detect cases of delayed infection.

### Phylogenetic analysis

Full DNA-A and DNA-B sequences from EuMV-Jal were compared with other New World and Old World begomoviruses available at the GenBank-NCBI database, using BLAST-N. The positions and sizes of EuMV-Jal open reading frames were predicted using EditSeq (DNASTAR Inc., Madison, WI). Paired alignments were obtained by the ClustalV and ClustalW methods in the MegAlign application of the Lasergene package (DNASTAR), using the default parameters. Neighbour-joining phylogenetic trees for EuMV DNA-A and DNA-B components were constructed using Mega 4.0 [66] with1,000 bootstrap replicates and pairwise evolutionary distances calculated with a maximum likelihood nucleotide substitution model. Trees were drawn to scale, with branch lengths in the same units as those of the evolutionary distances used to infer the phylogenetic tree.

### Recombination analysis

Detection of potential recombination breakpoints and recombinant sequences was carried out using the suite of recombination detection methods implemented in RDP software (29). A sequence alignment containing four EuMV isolates and closely related species (sharing >76% of nucleotide identity with EuMV-Jal) was used as input data for RDP. The analysis was performed using the default settings in all detection methods, with a Bonferroni corrected P-value cut-off of 0.05.

## Competing interests

The authors declare that they have not competing interests

## Authors' contributions

JGJ generated the infectious clones of EuMV-Jal, performed plant infections tests, carried out the phylogenetic analysis, and helped to prepare the manuscript. ABA collected isolates, cloned and sequence the viruses, analyzed the field data, and perform plant infection tests. BBH carried out the pseudorecombination experiments, and analyzed the experimentally infected plants. AAS helped in comparative sequence analyses, provided partial funding for the project's execution, and offered ideas and comments during manuscript preparation. CHZ carried out the recombination analysis, helped in plant infection tests. OMV provided the EuMV-YP clones and helped in plant infection tests with this virus. GFT collected isolates and helped to analyze the field data. GAA coordinated the project, carried out the comparative sequence analyses, secured funding for the project's execution, and prepared the manuscript. All authors read and approved the final manuscript.

## Supplementary Material

Additional file 1 **Supplemental Figure S1: Conserved elements upstream to s*Rep*HS elements**. (A) Comparisons of conserved modular arrangements (CMAs) composed by two *cis*-acting elements present in DNA-A of NW begomoviruses, and DNA-B of EuMV and relatives, respectively. The *CP*mRNA transcription start site of TGMV is indicated above the ERE-like motif. (B) Alignment of partially palindromic elements which are conserved in position relative to the s*Rep*HS element of ClLCrV and EuMV subclade members. The consensus of this symmetric element is indicated. Colors in boxes identify the distinct classes of s*Rep*HS according to their nucleotide sequence, and numbers indicate the length (in base pairs) of those elements. Click here for file
