# Supplementary figures and images for: Analysis of a new strain of Euphorbia mosaic virus with distinct replication specificity unveils a lineage of begomoviruses with short Rep sequences in the DNA-B intergenic region
Source: Virol J. 2010 Oct 19;7:275. doi: 10.1186/1743-422X-7-275 (PMC2974675; doi:10.1186/1743-422X-7-275)

## Slide 1
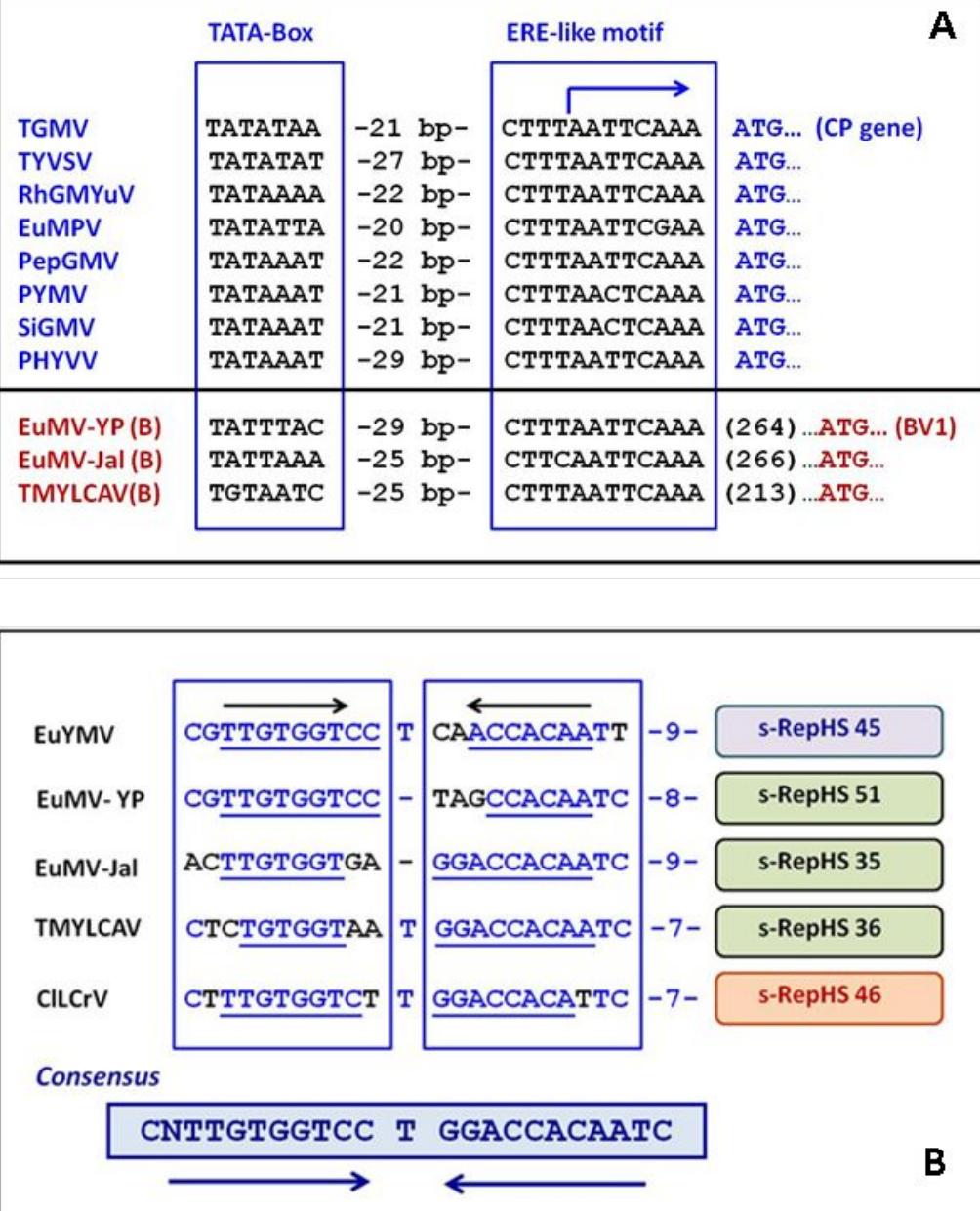

Supplement: Additional file 1 — Supplemental Figure S1: Conserved elements upstream to sRepHS elements. (A) Comparisons of conserved modular arrangements (CMAs) composed by two cis-acting elements present in DNA-A of NW begomoviruses, and DNA-B of EuMV and relatives, respectively. The CPmRNA transcription start site of TGMV is indicated above the ERE-like motif. (B) Alignment of partially palindromic elements which are conserved in position relative to the sRepHS element of ClLCrV and EuMV subclade members. The consensus of this symmetric element is indicated. Colors in boxes identify the distinct classes of sRepHS according to their nucleotide sequence, and numbers indicate the length (in base pairs) of those elements. [file 1743-422X-7-275-S1.PPT]
